# Supplementary material for: Iron deficiency causes aspartate-sensitive dysfunction in CD8+ T cells
Source: Nat Commun. 2025 Jun 20;16:5355. doi: 10.1038/s41467-025-60204-7 (PMC12181297; doi:10.1038/s41467-025-60204-7)
Supplement: Supplementary file 1 — Supplementary Information [file 41467_2025_60204_MOESM1_ESM.pdf]

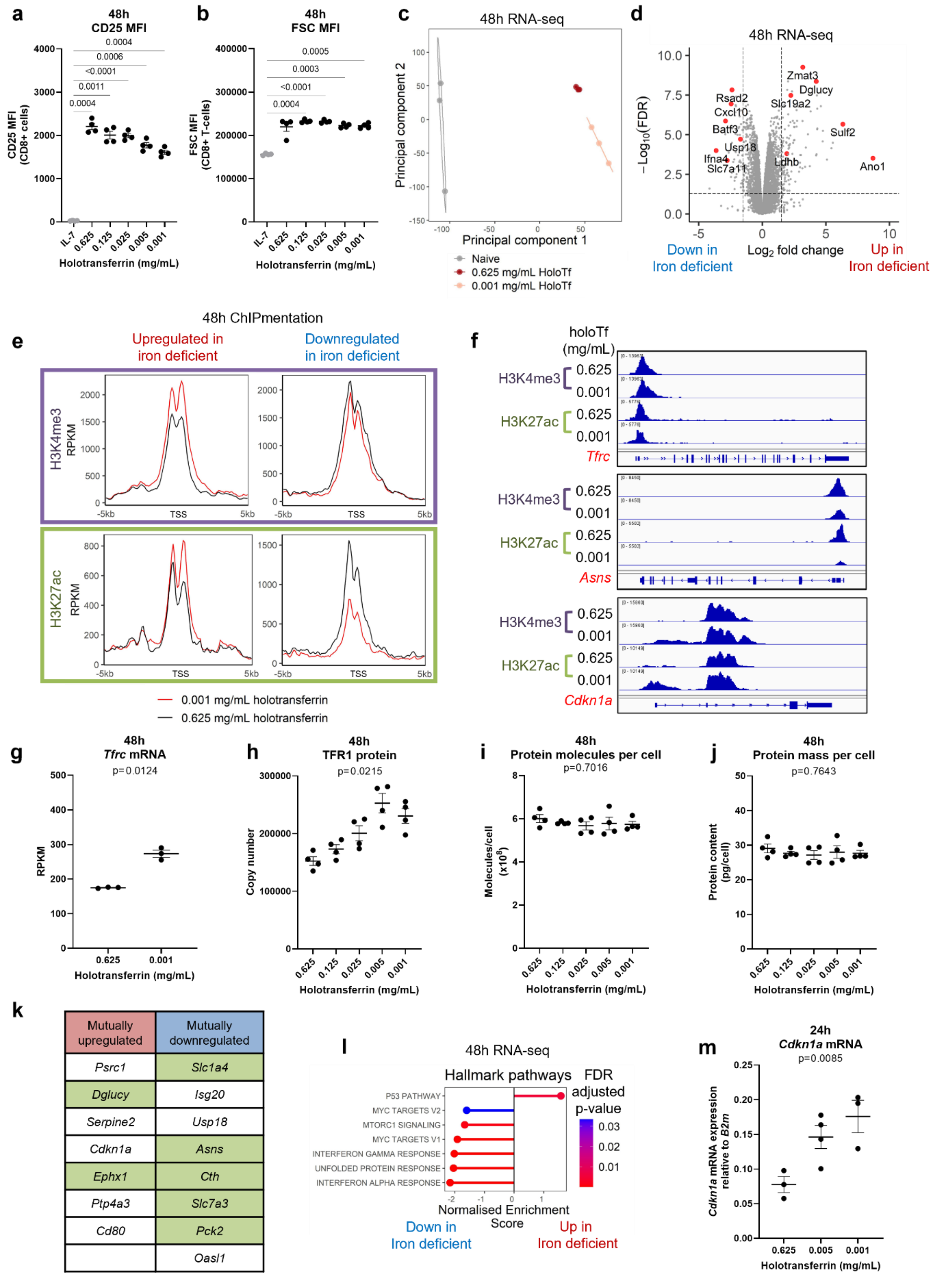

Supplementary Fig. 1| Iron restriction induces transcriptional and proteomic reprogramming. CD8+ T-cells were activated as described in Fig. 1a. Where comparisons between high and low iron conditions are made, the holotransferrin

concentrations used are 0.625 (high) and 0.001 (low) mg/mL. **(a)** CD25 MFI and **(b)** forward scatter (FSC) MFI, n=4. Naïve-like controls cells were cultured in IL-7 (5 ng/mL). **(c)** PCA of RNA-seq samples, n=4. **(d)** Volcano plot of RNA-seq with significance thresholds of false discovery rate (FDR) < 0.05 and $\log_2$|fold change (FC)| > 1.5, n=4. A Benjamini-Hochberg adjustment was used to correct for multiple testing. **(e)** H3K4me3 and H3K27ac enrichment measured using ChIPmentation at transcription start sites (TSSs) of genes identified to be up or downregulated by iron-deficiency in the RNA-seq, n=1. RPKM = reads per kilobase per million mapped reads. **(f)** Genome tracks for *Tfrc*, *Asns* and *Cdkn1*a showing H3K4me3 and H3K27ac peaks under high and low iron conditions, n=1. *Tfrc*/TFR1 **(g)** mRNA and **(h)** protein expression, n=4. **(i)** protein molecules and **(j)** protein mass per cell measured via protein-MS, n=4. **(k)** Genes and proteins mutually up or downregulated in low iron conditions, n=4. Metabolic genes are in green. **(l)** Hallmark gene set enrichment analysis (GSEA) for the RNA-seq, n=4. A Benjamini-Hochberg adjustment was used to correct for multiple testing. **(m)** *Cdkn1a* mRNA expression assessed by qPCR at 24h, n=4. Data is mean ± SEM where each datapoint per condition denotes cells from independent donor mice. Statistics are: **(a-b)** sampled matched one-way ANOVAs with the Geisser-Greenhouse correction and Tukey's test for multiple comparisons, **(g)** paired t-test; **(h-j, m)** sample matched one-way ANOVAs with the Geisser-Greenhouse correction. Source data are provided as a Source Data file.

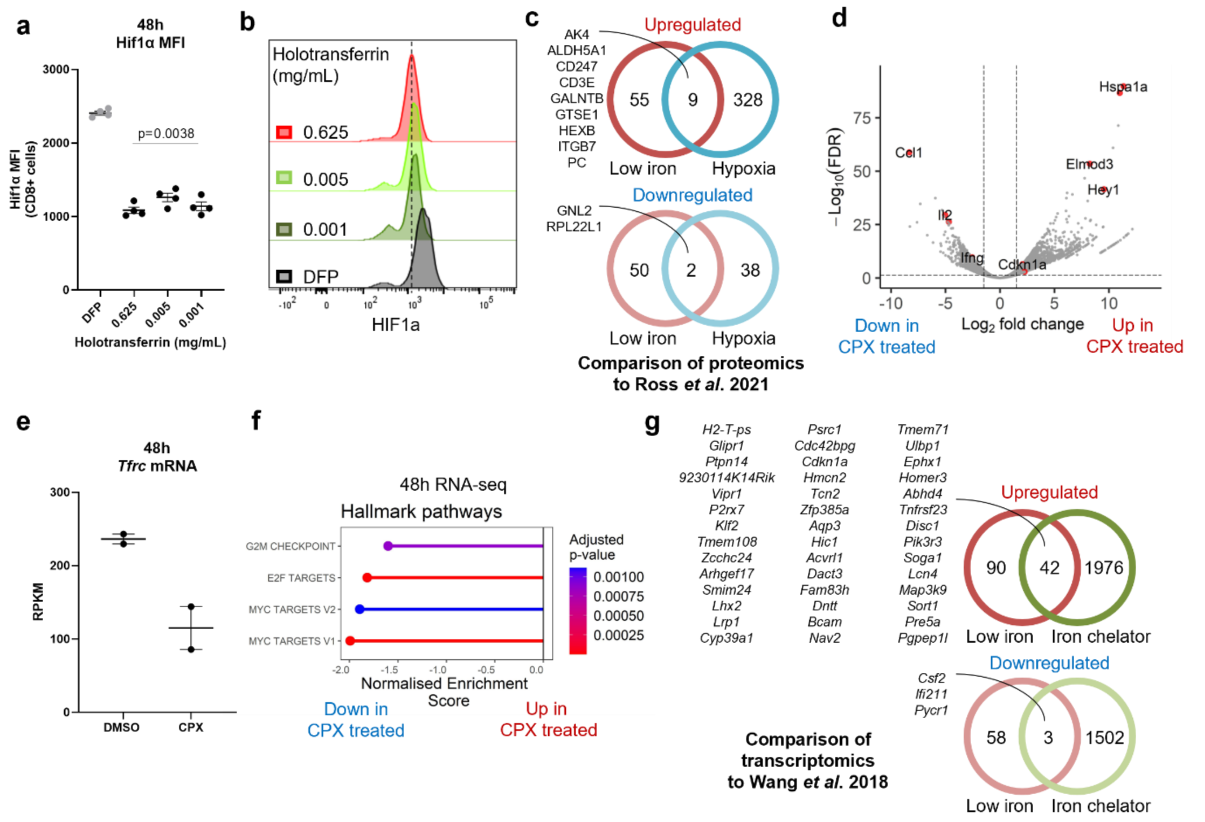

Supplementary Fig. 2| Iron deficiency induces a response distinct from either hypoxia or iron chelators. CD8+ T-cells were activated as described for Fig. 1a. **(a-b)** HIF1α MFI, n=4. Controls were treated for 4h with deferiprone (DFP; 75 μM). **(c)** Venn diagrams of proteins mutually up or downregulated in response to iron deficient conditions and hypoxia (1% O_2 relative to normoxia at 18% O_2) in CD8+ T-cells. Hypoxia data are derived from Ross *et al*[28]. **(d-g)** Raw data was analysed from Wang *et al*[29] (GSE84702) where CD4+ T-cells were cultured in Th1 polarising conditions for 5 days and then treated with DMSO control or the iron chelator, ciclopirox (CPX; 50 μM) for 4 hours. **(d)** Volcano plot where significance thresholds of FDR < 0.05 and $\log_2$|fold change| > 1.5 were applied. **(e)** *Tfrc* mRNA expression. RPKM = reads per kilobase of transcript per million mapped reads. **(f)** Hallmark gene set enrichment analysis. A Benjamini-Hochberg adjustment was used to correct for multiple testing. **(g)** Venn diagrams of transcripts mutually up or downregulated in response to iron deficient conditions and CPX iron chelation. Data is mean ± SEM where each datapoint per condition denotes cells from independent donor mice. Statistics are: **(a)** one-way ANOVA with the Geisser-Greenhouse correction. Source data are provided as a Source Data file.

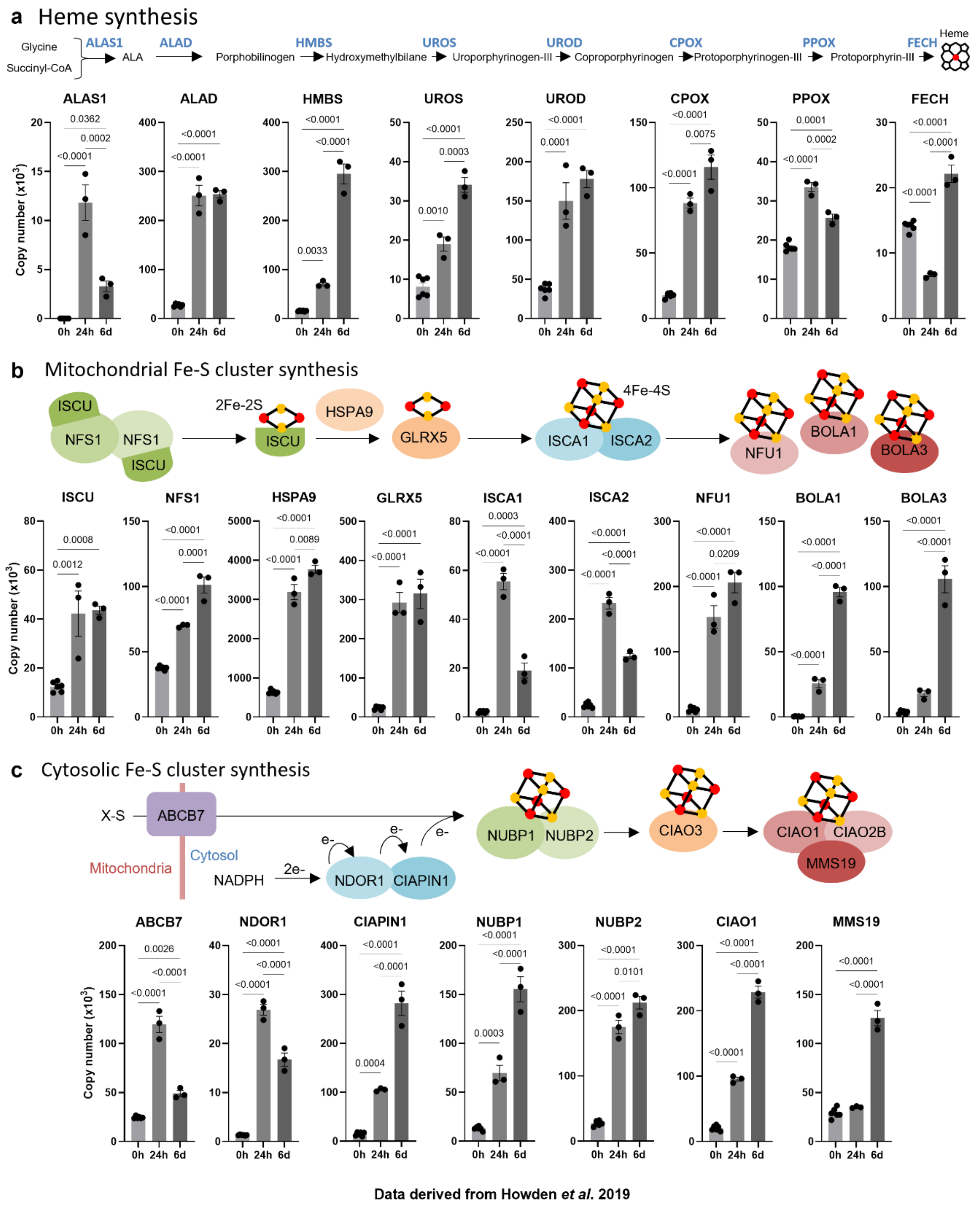

Supplementary Fig. 3| Heme and Fe-S cluster synthesis proteins are induced following CD8+ T cell activation. Data derived from Howden *et al*[14]. Protein copy numbers for proteins involved in **(a)** heme synthesis, **(b)** mitochondrial Fe-S cluster synthesis and **(c)** cytosolic Fe-S cluster synthesis. n=6 at 0h, n=3 at 24h and 6d. Data is mean ± SEM where each datapoint per condition denotes cells from independent donor mice. Statistics are: **(a-c)** ordinary one-way ANOVAs with multiple comparisons using Tukey's correction. Source data are provided as a Source Data file.

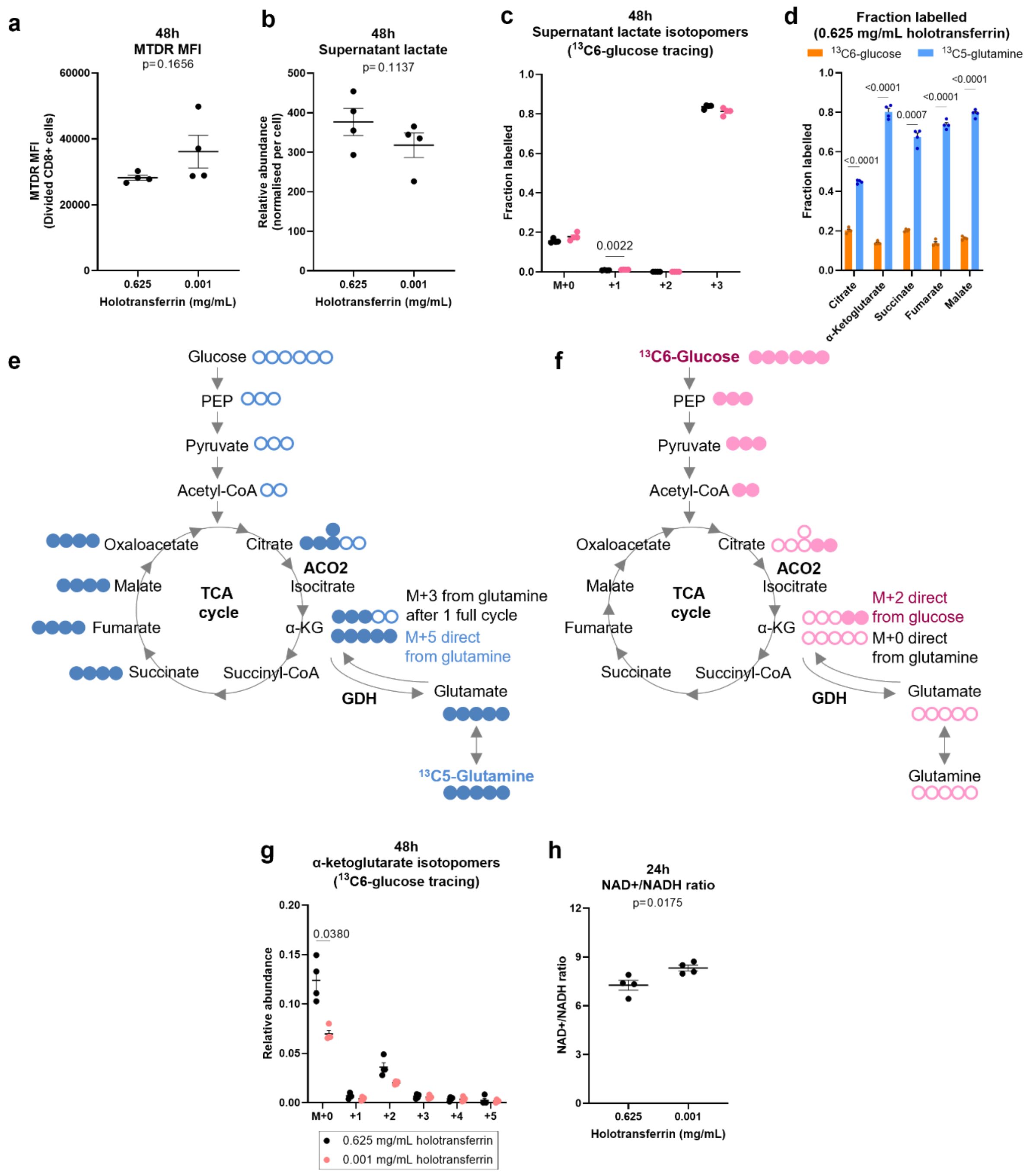

Supplementary Fig. 4| Iron-deficiency alters CD8+ T-cell mitochondrial metabolism. CD8+ T-cells were activated as described in Fig. 1a. For the ^{13}C6-glucose and ^{13}C5-glutamine tracing experiments, T-cells were activated for 24h and then incubated in media containing ^{13}C6-glucose or ^{13}C5-glutamine for a further 24h. **(a)** Mitotracker deep red (MTDR) MFI, n=4. **(b)** Lactate measured in cell supernatant normalised by spiked in glutaric acid and cell number. **(c)** Fraction of lactate labelled by ^{13}C6-glucose in cell supernatants. **(d)** Metabolic fraction labelled by either ^{13}C6-glucose or ^{13}C5-glutamine in high iron conditions (0.625 mg/mL holotransferrin), n=4. Schematic

of tracing into α-KG from **(e)** ^{13}C5-glutamine and **(f)** ^{13}C6-glucose. Blue filled circles indicate ^{13}C labelled atoms from ^{13}C5-glutamine. Pink filled circles indicate ^{13}C labelled atoms from ^{13}C6-glucose. Empty circles indicate unlabelled carbon atoms. **(g)** Relative abundance of α-KG mass isotopomers from ^{13}C-glucose tracing calculated as the fraction labelled multiplied by the raw glutaric acid normalised abundance, n=4. **(h)** NAD+/NADH ratio, n=4. Data is mean ± SEM where each datapoint per condition denotes cells from independent donor mice. Statistics are: **(a, b, h)** matched two-tailed t-test; **(c, g)** matched two-way ANOVAs with the Geisser-Greenhouse correction and the Sidak correction for multiple comparisons. **(d)** two-way ANOVA with sample matching between metabolites but not between the two carbon tracers. Source data are provided as a Source Data file.

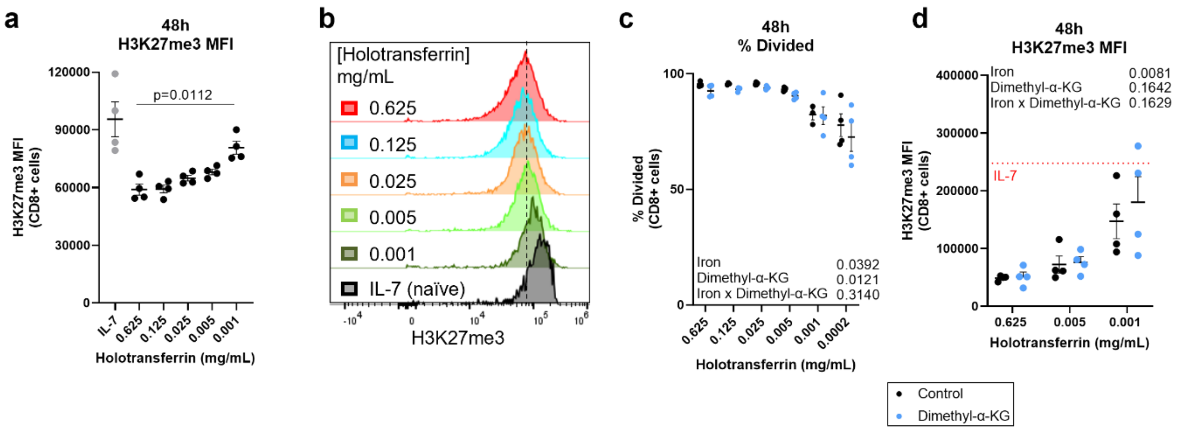

Supplementary Fig. 5| H3K27me3 accumulates in iron deprived CD8+ T-cells by 48h. CD8+ T-cells were activated as described in Fig. 1a. **(a-b)** H3K27me3 MFI measured at 48h, n=4. "Naïve" controls cells were cultured in IL-7 (5 ng/mL). **(c)** Percentage divided cells and **(d)** H3K27me3 MFI of cells cultured with or without dimethyl-α-KG (1 mM), n=4. Data is mean ± SEM where each datapoint per condition denotes cells from independent donor mice. Histograms are normalised to mode. Statistics are: **(a)** matched one-way ANOVA with the Geisser-Greenhouse correction; **(c)** matched mixed effect analysis with the Geisser-Greenhouse correction; **(d)** two-way ANOVA with the Geisser-Greenhouse correction. Source data are provided as a Source Data file.

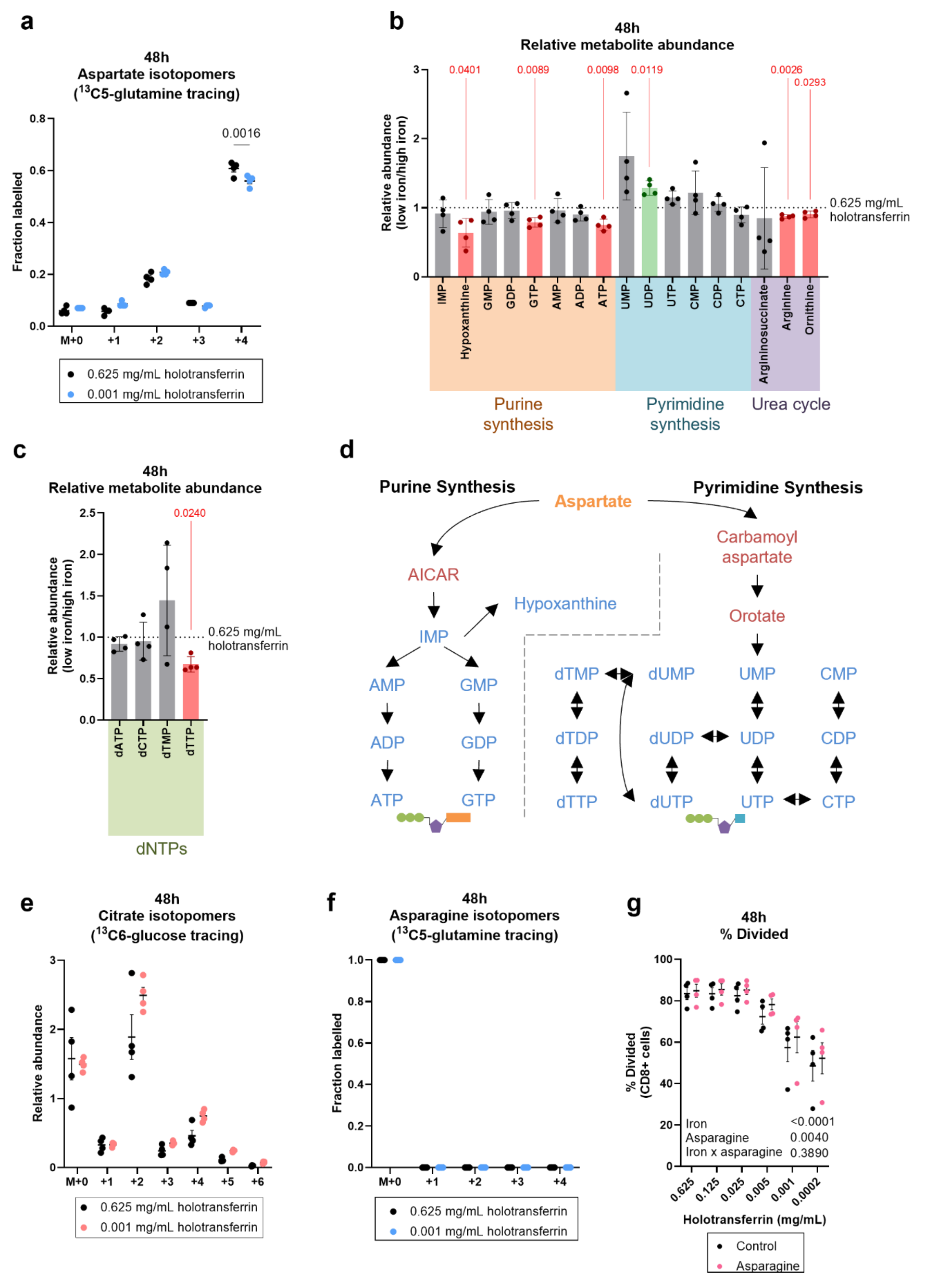

Supplementary Fig. 6| Iron deficient T-cells suppress aspartate utilising pathways. Isolated CD8+ T-cells were activated as described in Fig. 1a. For the 13C6-glucose and 13C5-glutamine tracing experiments, T-cells were activated for 24h and then incubated in

media containing $^{13}C6$-glucose or $^{13}C5$-glutamine for a further 24h. **(a)** Aspartate and isotopomers labelled from $^{13}C5$-glutamine, n=4. M+1/2/3/4 indicate isotopomers derived from glutamine. **(b-c)** Relative metabolite abundance of T-cells in low iron (0.001 mg/mL holotransferrin) versus high iron (0.625 mg/mL holotransferrin) normalised to spiked in glutaric acid, n=4. P-values are shown in red. **(d)** Aspartate is incorporated into ribonucleotides which can be interconverted between mono, di and tri-phosphorylated forms or converted to deoxy-ribonucleotides. **(e)** Citrate isotopomers labelled from $^{13}C6$-glucose, n=4. M+1/2/3/4/5/6 indicate isotopomers derived from glucose. **(f)** Asparagine isotopomers labelled from $^{13}C5$-glutamine, n=4. M+1/2/3/4 indicate isotopomers derived from glutamine. **(g)** Division assessed with cell trace violet (CTV) with or without asparagine (100 μM), n=4. Data is mean ± SEM where each datapoint per condition denotes cells from independent donor mice. Statistics are: **(a, e-f)** matched two-way ANOVA with the Geisser-Greenhouse correction and the Fisher's least significant difference (LSD) test for multiple comparisons; **(b-c)** matched t-tests between 0.625 and 0.001 mg/mL holotransferrin conditions for each metabolite; **(g)** two-way ANOVA with the Geisser-Greenhouse correction. Source data are provided as a Source Data file.

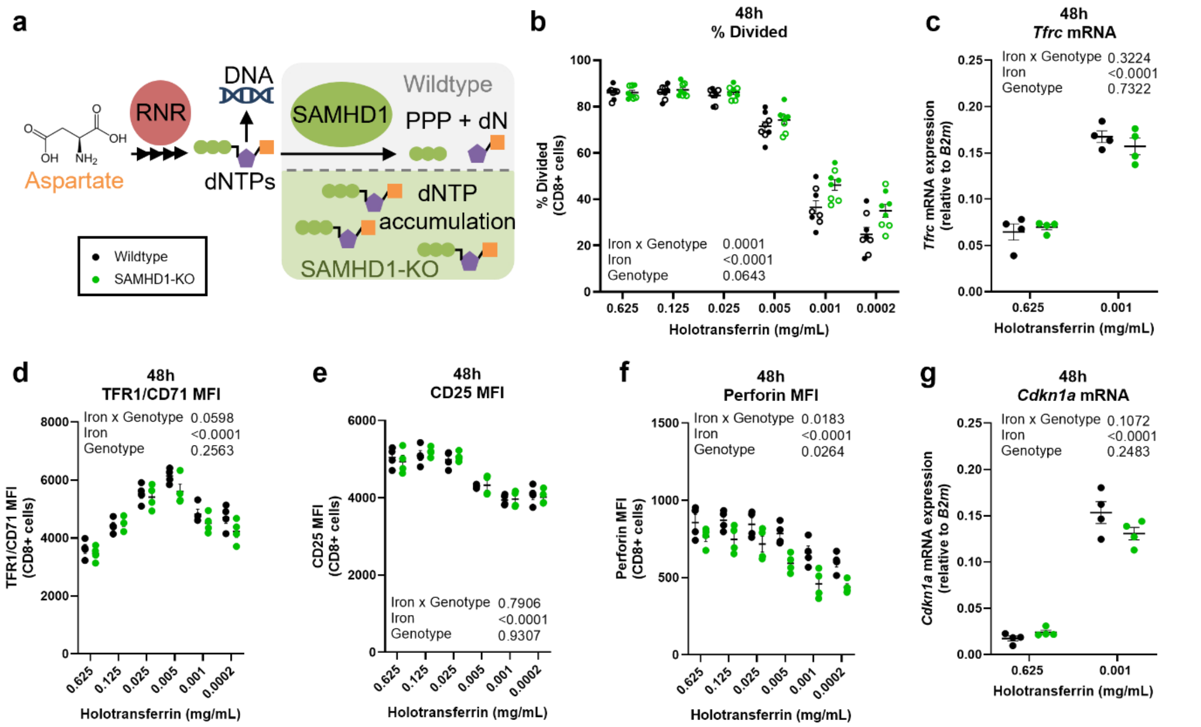

Supplementary Fig. 7| SAMHD1-KO T-cells show resistance to iron-deficiency suppressed proliferation. T-cells were isolated from SAMHD1-KO mice and wildtype littermate controls and were activated as described in Fig. 1a. **(a)** Ribonucleotide reductase (RNR) enables dNTP production, SAMHD1 degrades dNTPs. SAMHD1-KO should result in dNTP accumulation. **(b)** Percentage divided cells measured using cell trace violet (CTV). Data from independent experiments denoted by different symbols, n=8. *Tfrc*/TFR1/CD71 **(c)** mRNA and **(d)** surface protein MFI, n=4. **(e)** CD25 and **(f)** perforin MFI, n=4. **(g)** *Cdkn1a* mRNA expression by qPCR, n=4. Data is mean ± SEM where each datapoint per condition denotes cells from independent donor mice. Statistics are: **(b-g)** sample matched two-way ANOVAs with the Geisser-Greenhouse correction applied for **(d-f)**. Source data are provided as a Source Data file.

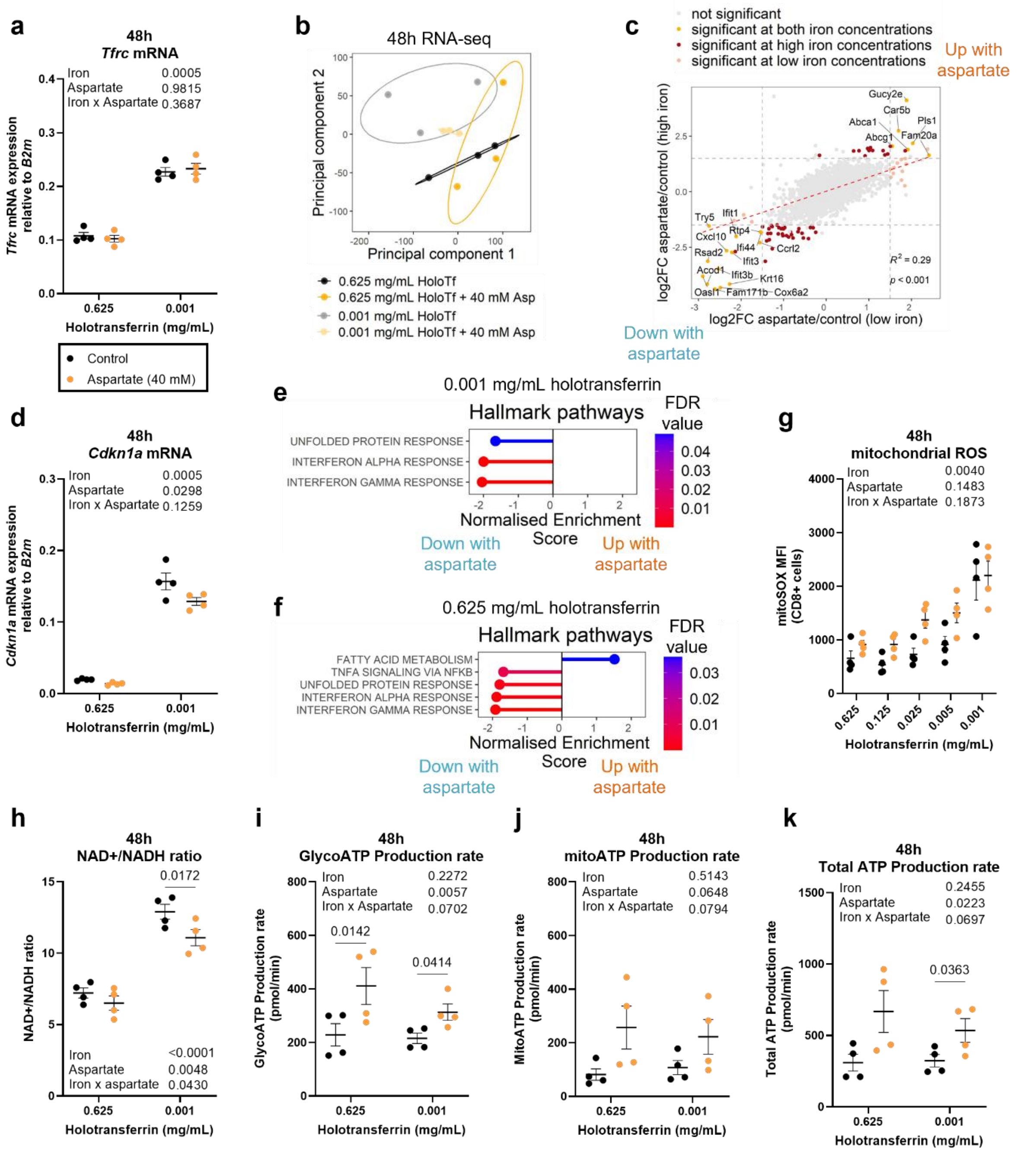

Supplementary Fig. 8| Aspartate has limited impact on cellular transcription but promotes a more metabolic state in CD8+ T-cells. CD8+ T-cells were activated as described in Fig. 1a with or without aspartate (40 mM). **(a)** *Tfrc* mRNA expression by qPCR, n=4. **(b)** RNA-seq PCA, n=4. **(c)** Correlation plot comparing the $\log_2$(fold change (FC)) between aspartate and control conditions at either low iron or high iron conditions, n=4. Significance thresholds of $|\log_2 FC| > 1.5$ and a false discovery rate (FDR) < 0.05 were applied. A Benjamini-Hochberg adjustment was used to correct for multiple testing. **(d)** *Cdkn1a* mRNA expression, n=4. Gene set enrichment analysis (GSEA) of aspartate treated

CD8+ T-cells versus control in **(e)** low iron conditions or **(f)** high iron conditions, n=4. A Benjamini-Hochberg adjustment was used to correct for multiple testing. **(g)** mROS MFI, n=4. **(h)** NAD+/NADH ratio, n=4. **(i)** Glycolytic, **(j)** mitochondrial and **(k)** total ATP production rate measured using the ATP rate seahorse kit, n=4. Data is mean ± SEM where each datapoint per condition denotes cells from independent donor mice. Statistics are: **(a, d, g-k)** two-way ANOVAs with the Geisser-Greenhouse correction with Sidak's test for multiple comparisons for **(h-k)**. **(c)** two-sided Pearson correlation R^2 value. Source data are provided as a Source Data file.

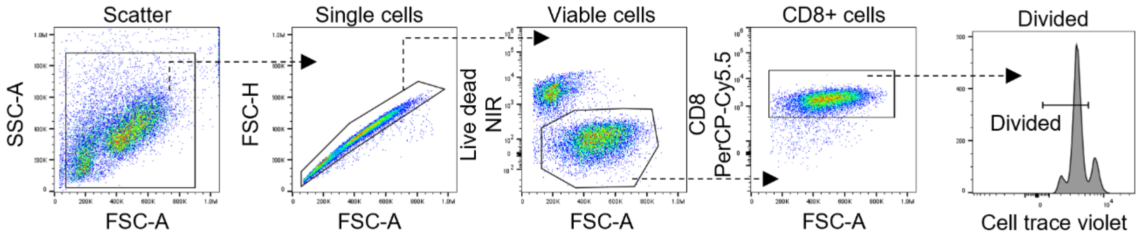

Supplementary Fig. 9| Representative gating scheme of *in vitro* activated CD8+ T-cells. *In vitro* activated CD8+ T-cells stimulated with 5 µg/mL plate bound α-CD3, 1 µg/mL α-CD28 and 50 U/mL IL-2.
