# Supplementary material for: Iron deficiency causes aspartate-sensitive dysfunction in CD8+ T cells
Source: Nat Commun. 2025 Jun 20;16:5355. doi: 10.1038/s41467-025-60204-7 (PMC12181297; doi:10.1038/s41467-025-60204-7)
Supplement: Supplementary file 2 — Description of Additional Supplementary Files [file 41467_2025_60204_MOESM2_ESM.pdf]

Description of Additional Supplementary Files

Supplementary Data 1: RNAseq of iron deficient and iron replete T-cells

Supplementary Data 2: protein MS of iron deficient and iron replete T-cells

Supplementary Data 3: RNAseq of iron deficient and iron replete T-cells with and without aspartate
