# Supplementary material for: Iron deficiency causes aspartate-sensitive dysfunction in CD8+ T cells
Source: Nat Commun. 2025 Jun 20;16:5355. doi: 10.1038/s41467-025-60204-7 (PMC12181297; doi:10.1038/s41467-025-60204-7)
Supplement: Supplementary file 6 — Reporting Summary [file 41467_2025_60204_MOESM6_ESM.pdf]

nature portfolio

Corresponding author(s): Hal Drakesmith

Last updated by author(s): Apr 9, 2025

# Reporting Summary

Nature Portfolio wishes to improve the reproducibility of the work that we publish. This form provides structure for consistency and transparency in reporting. For further information on Nature Portfolio policies, see our Editorial Policies and the Editorial Policy Checklist.

## Statistics

For all statistical analyses, confirm that the following items are present in the figure legend, table legend, main text, or Methods section.

| n/a | Confirmed | |
|---|---|---|
| ☐ | ☒ | The exact sample size (*n*) for each experimental group/condition, given as a discrete number and unit of measurement |
| ☐ | ☒ | A statement on whether measurements were taken from distinct samples or whether the same sample was measured repeatedly |
| ☐ | ☒ | The statistical test(s) used AND whether they are one- or two-sided<br>*Only common tests should be described solely by name; describe more complex techniques in the Methods section.* |
| ☒ | ☐ | A description of all covariates tested |
| ☐ | ☒ | A description of any assumptions or corrections, such as tests of normality and adjustment for multiple comparisons |
| ☐ | ☒ | A full description of the statistical parameters including central tendency (e.g. means) or other basic estimates (e.g. regression coefficient) AND variation (e.g. standard deviation) or associated estimates of uncertainty (e.g. confidence intervals) |
| ☐ | ☒ | For null hypothesis testing, the test statistic (e.g. *F*, *t*, *r*) with confidence intervals, effect sizes, degrees of freedom and *P* value noted<br>*Give P values as exact values whenever suitable.* |
| ☒ | ☐ | For Bayesian analysis, information on the choice of priors and Markov chain Monte Carlo settings |
| ☒ | ☐ | For hierarchical and complex designs, identification of the appropriate level for tests and full reporting of outcomes |
| ☐ | ☒ | Estimates of effect sizes (e.g. Cohen's *d*, Pearson's *r*), indicating how they were calculated |

*Our web collection on statistics for biologists contains articles on many of the points above.*

## Software and code

Policy information about availability of computer code

| | |
|---|---|
| Data collection | Next generation sequencing data was collected via the Illumina system.<br>Flow cytometry data was acquired using attune NxT software. |

Data analysis

For the RNA-seq: Read quality was assessed using FastQC (version 0.11.9). Read alignment was conducted using RNA-star (version 2.7.3a) to the Mus Musculus genome (mm39) and features were annotated using FeatureCounts from the subread package (version 2.0.0 and 2.0.3). Differential gene expression analysis was conducted using the R package, EdgeR (version 3.38.4). Gene set enrichment analysis was completed using FGSEA R package (version 1.22.0) and Hallmark pathways.

For the proteomics: Quantification of reporter ions was completed using spectronaut (Biognosys; Spectronaut 14.10.201222.47784) in library-free (directDIA) mode. Data filtering and protein copy number quantification were performed in the Perseus software package, version 1.6.6.0. GSEA was conducted using the FGSEA R package (version 1.22.0) and Hallmark pathways.

For the ChIPmentation: Data was analysed using the SeqNado analysis pipeline (https://github.com/alsmith151/SeqNado).

For the metabolite-MS: Data processing was completed using MATLAB or the using the FreeStyle 1.6 software and the Genesis peak detection algorithm with its default settings.

For flow cytometry: Data was analysed using FlowJo (version 10.8.1-10.10.0)

General: Data was analysed using Prism (version 10) and excel 2016

For manuscripts utilizing custom algorithms or software that are central to the research but not yet described in published literature, software must be made available to editors and reviewers. We strongly encourage code deposition in a community repository (e.g. GitHub). See the Nature Portfolio guidelines for submitting code & software for further information.

# Data

Policy information about availability of data

All manuscripts must include a data availability statement. This statement should provide the following information, where applicable:

- Accession codes, unique identifiers, or web links for publicly available datasets
- A description of any restrictions on data availability
- For clinical datasets or third party data, please ensure that the statement adheres to our policy

The datasets associated with this paper can be found within the paper or have been submitted to GEO (GSE251962, GSE251963, GSE251964, GSE292414), the proteomeXchange (PXD047814) or MetaboLights (Megan Teh: Raw LC-MS data). They are available at the following links:

GSE251962 - https://www.ncbi.nlm.nih.gov/geo/query/acc.cgi?acc=GSE251962

GSE251963 - https://www.ncbi.nlm.nih.gov/geo/query/acc.cgi?acc=GSE251963

GSE251964 - https://www.ncbi.nlm.nih.gov/geo/query/acc.cgi?acc=GSE251964

GSE292414 - https://www.ncbi.nlm.nih.gov/geo/query/acc.cgi?acc=GSE292414

PXD047814 - https://www.ebi.ac.uk/pride/archive/projects/PXD047814

Megan Teh: Raw LC-MS data - https://metabolights-labs.org/u/christopher_lee_millington/h/megan-teh-raw-lc-ms-data

MSV000097525 - https://massive.ucsd.edu/ProteoSAFe/dataset.jsp?task=ec2369d9e92b40acabce8f292df2cc2c)

MSV000097526 - https://massive.ucsd.edu/ProteoSAFe/dataset.jsp?task=db3cd5a35e034026ad6d73b793eea3ee

MSV000097527 - https://massive.ucsd.edu/ProteoSAFe/dataset.jsp?task=dbd7ebd8ec454ea1ad0312b8b90ba981

MSV000097528 - https://massive.ucsd.edu/ProteoSAFe/dataset.jsp?task=bf2885289e684df2bcfb3e04854a6cc0

# Research involving human participants, their data, or biological material

Policy information about studies with human participants or human data. See also policy information about sex, gender (identity/presentation), and sexual orientation and race, ethnicity and racism.

Reporting on sex and gender

*Use the terms sex (biological attribute) and gender (shaped by social and cultural circumstances) carefully in order to avoid confusing both terms. Indicate if findings apply to only one sex or gender; describe whether sex and gender were considered in study design; whether sex and/or gender was determined based on self-reporting or assigned and methods used.*
*Provide in the source data disaggregated sex and gender data, where this information has been collected, and if consent has been obtained for sharing of individual-level data; provide overall numbers in this Reporting Summary. Please state if this information has not been collected.*
*Report sex- and gender-based analyses where performed, justify reasons for lack of sex- and gender-based analysis.*

Reporting on race, ethnicity, or other socially relevant groupings

*Please specify the socially constructed or socially relevant categorization variable(s) used in your manuscript and explain why they were used. Please note that such variables should not be used as proxies for other socially constructed/relevant variables (for example, race or ethnicity should not be used as a proxy for socioeconomic status).*
*Provide clear definitions of the relevant terms used, how they were provided (by the participants/respondents, the*

| | |
|---|---|
| | *researchers, or third parties), and the method(s) used to classify people into the different categories (e.g. self-report, census or administrative data, social media data, etc.)* *Please provide details about how you controlled for confounding variables in your analyses.* |
| Population characteristics | *Describe the covariate-relevant population characteristics of the human research participants (e.g. age, genotypic information, past and current diagnosis and treatment categories). If you filled out the behavioural & social sciences study design questions and have nothing to add here, write "See above."* |
| Recruitment | *Describe how participants were recruited. Outline any potential self-selection bias or other biases that may be present and how these are likely to impact results.* |
| Ethics oversight | *Identify the organization(s) that approved the study protocol.* |

Note that full information on the approval of the study protocol must also be provided in the manuscript.

# Field-specific reporting

Please select the one below that is the best fit for your research. If you are not sure, read the appropriate sections before making your selection.

☒ Life sciences ☐ Behavioural & social sciences ☐ Ecological, evolutionary & environmental sciences

For a reference copy of the document with all sections, see nature.com/documents/nr-reporting-summary-flat.pdf

# Life sciences study design

All studies must disclose on these points even when the disclosure is negative.

| | |
|---|---|
| Sample size | Statistical methods were not used to determine the sample size. Sample sizes (typically n=4) were chosen based on similar studies with similar techniques where statistical analysis was assessed to be reasonable (Frost et al, 2021; Gudgeon et al, 2022; Jenkins et al, 2023).<br><br>Biological effects of interest were found to be consistent across replicates. |
| Data exclusions | Two data points were excluded in figure S4C. One sample for each of the iron conditions 0.125 mg/mL holotransferrin and 0.001 mg/mL holotransferrin in the control condition had viabilities lower than 40% and were thus excluded. Viabilities for our CD8+ T-cell culture are typically ~70% and therefore samples with viabilities <40% are highly unusual and suggest an issue with the cell culture beyond the treatment conditions.<br><br>No other data was excluded. |
| Replication | Proteomics, RNA-seq and metabolomic-MS were all conducted with 4 biological replicates to ensure that changes were consistent across independent samples. Due to cost constraints, the proteomics, RNA-seq and glucose/glutamine tracing experiments were only conducted once. However, gene expression data was found to be relatively consistent between the RNA-seq and protein-MS. Similarly, absolute metabolite abundances measured by mass spectrometry typically repeated across 2-3 metabolite-MS experiments.<br><br>ChIPmentation data for H3K4me3 and H3K27ac represents a single replicate but is consistent with data collected by RNA-seq. ChIPmentation data for H3K27me3 represents 3 biological replicates run in 1 experiment - however, the data was consistent with flow cytometry measurements of H3K27me3 which were repeated at least 3 times.<br><br>All other experiments were repeated 2-3 times with the exceptions of 6i, 6l, S2a-b, S5d, S6f, S8h |
| Randomization | Randomisation is not relevant to this study. All independent biological samples were treated with all iron conditions used for a particular experiment. |
| Blinding | Blinding was not utilised in this study. |

# Reporting for specific materials, systems and methods

We require information from authors about some types of materials, experimental systems and methods used in many studies. Here, indicate whether each material, system or method listed is relevant to your study. If you are not sure if a list item applies to your research, read the appropriate section before selecting a response.

## Materials & experimental systems

| n/a | Involved in the study | |
|---|---|---|
| [ ] | [x] | Antibodies |
| [x] | [ ] | Eukaryotic cell lines |
| [x] | [ ] | Palaeontology and archaeology |
| [ ] | [x] | Animals and other organisms |
| [x] | [ ] | Clinical data |
| [x] | [ ] | Dual use research of concern |
| [x] | [ ] | Plants |

## Methods

| n/a | Involved in the study | |
|---|---|---|
| [ ] | [x] | ChIP-seq |
| [ ] | [x] | Flow cytometry |
| [x] | [ ] | MRI-based neuroimaging |

# Antibodies

Antibodies used

CD25-APC (Biolegend, PC61, 102011, 1:200), CD25-PE-Cy7 (Biolegend, PC61, 102015, 1:200), CD44-BV605 (Biolegend, IM7, 103047), CD71-FITC (Biolegend, RI7217, 113805, 1:400), CD71-APC (Biolegend, RI7217, 113819, 1:200), CD71-PE-Cy7 (Biolegend, RI7217, 113811, 1:200), CD71-PE (Biolegend, RI7217, 113807, 1:200), CD8a-FITC (Biolegend, 53-6.7, 100705, 1:400), CD8a-PE (Biolegend, 53-6.7, 100707, 1:200), CD8a-PerCP-Cy5.5 (Biolegend, 53-6.7, 100733, 1:200), CD98-APC (Biolegend, RL388, 128211, 1:200), CD107a-FITC (Biolegend, 1D4B, 121606, 1:400), GZMB-FITC (Biolegend, GB11, 515403, 1:100), H3K27me3-AF488 (Cell Signalling, C36B11, 5499S, 1:50), IFNg-Pe-Cy7 (Biolegend, XMG1.2, 505825, 1:200), IL-2-PE (Biolegend, JES6-5H4, 503807, 1:200), Perforin-PE (Biolegend, S16009B, 154405, 1:200), pS6-AF647 (Cell Signalling, D57.2.2E, 14733S, 1:50), TNFa-FITC (Biolegend, MP6-XT22, 506603, 1:400), H3K27ac (Diagenode, polyclonal, C15410196), H3K4me3 (Diagenode, polyclonal, C15410003), H3K27me3 (Sigma Aldrich, polyclonal, 7-449)

Validation

The antibodies described are well validated for either flow cytometry or ChiP-seq by the manufacturers (https://www.biolegend.com/en-us, https://www.diagenode.com/en, https://www.sigmaaldrich.com/).

Antibodies being utilised for the first time by us were tested by flow cytometry with fluorescence minus one controls.

# Animals and other research organisms

Policy information about studies involving animals; ARRIVE guidelines recommended for reporting animal research, and Sex and Gender in Research

Laboratory animals

OT-I mice on a C57BL/6 background and SAMHD1-KO mice, male and female, age between 6-20 weeks

Wild animals

This study did not involve wild animals

Reporting on sex

Data reporting on sex differences has not been collected. Female and male mice were both used in this study and similar biological effects were observed in samples collected from male and female mice. Individual experiments were typically conducted using animals of the same sex; however, effects were found to replicate regardless of the sex of the cells in subsequent experiments.

Field-collected samples

This study did not involve samples collected in the field

Ethics oversight

Animal work was completed under the authority of UK home office project and personal licenses granted under the Animals (Scientific Procedures) Act (ASPA) 1986.

Note that full information on the approval of the study protocol must also be provided in the manuscript.

# Plants

Seed stocks

*Report on the source of all seed stocks or other plant material used. If applicable, state the seed stock centre and catalogue number. If plant specimens were collected from the field, describe the collection location, date and sampling procedures.*

Novel plant genotypes

*Describe the methods by which all novel plant genotypes were produced. This includes those generated by transgenic approaches, gene editing, chemical/radiation-based mutagenesis and hybridization. For transgenic lines, describe the transformation method, the number of independent lines analyzed and the generation upon which experiments were performed. For gene-edited lines, describe the editor used, the endogenous sequence targeted for editing, the targeting guide RNA sequence (if applicable) and how the editor was applied.*

Authentication

*Describe any authentication procedures for each seed stock used or novel genotype generated. Describe any experiments used to assess the effect of a mutation and, where applicable, how potential secondary effects (e.g. second site T-DNA insertions, mosiacism, off-target gene editing) were examined.*

# ChIP-seq

## Data deposition

- [x] Confirm that both raw and final processed data have been deposited in a public database such as GEO.
- [x] Confirm that you have deposited or provided access to graph files (e.g. BED files) for the called peaks.

Data access links
*May remain private before publication.*

GSE251964 - https://www.ncbi.nlm.nih.gov/geo/query/acc.cgi?acc=GSE251964
GSE292414 - https://www.ncbi.nlm.nih.gov/geo/query/acc.cgi?acc=GSE292414

Files in database submission

Mtc77_H3K27ac_1_1.fastq.gz
Mtc77_H3K27ac_2_1.fastq.gz
Mtc77_H3K4me3_1_1.fastq.gz
Mtc77_H3K4me3_2_1.fastq.gz
Mtc77_H3K27ac_1_2.fastq.gz
Mtc77_H3K27ac_2_2.fastq.gz
Mtc77_H3K4me3_1_2.fastq.gz
Mtc77_H3K4me3_2_2.fastq.gz
Mtc77_H3K27ac_1_H3K27ac.bigWig
Mtc77_H3K27ac_2_H3K27ac.bigWig
Mtc77_H3K4me3_1_H3K4me3.bigWig
Mtc77_H3K4me3_2_H3K4me3.bigWig
Mtc77_H3K27ac_1_H3K27ac.bed
Mtc77_H3K27ac_2_H3K27ac.bed
Mtc77_H3K4me3_1_H3K4me3.bed
Mtc77_H3K4me3_2_H3K4me3.bed

CM-mtc91-high-iron-1-H3K27me3_CM-mtc91-high-iron-1-H3K27me3_1.fastq.gz
CM-mtc91-high-iron-1-H3K27me3_CM-mtc91-high-iron-1-H3K27me3_2.fastq.gz
CM-mtc91-high-iron-2-H3K27me3_CM-mtc91-high-iron-2-H3K27me3_1.fastq.gz
CM-mtc91-high-iron-2-H3K27me3_CM-mtc91-high-iron-2-H3K27me3_2.fastq.gz
CM-mtc91-high-iron-3-H3K27me3_CM-mtc91-high-iron-3-H3K27me3_1.fastq.gz
CM-mtc91-high-iron-3-H3K27me3_CM-mtc91-high-iron-3-H3K27me3_2.fastq.gz
CM-mtc91-low-iron-1-H3K27me3_CM-mtc91-low-iron-1-H3K27me3_1.fastq.gz
CM-mtc91-low-iron-1-H3K27me3_CM-mtc91-low-iron-1-H3K27me3_2.fastq.gz
CM-mtc91-low-iron-2-H3K27me3_CM-mtc91-low-iron-2-H3K27me3_1.fastq.gz
CM-mtc91-low-iron-2-H3K27me3_CM-mtc91-low-iron-2-H3K27me3_2.fastq.gz
CM-mtc91-low-iron-3-H3K27me3_CM-mtc91-low-iron-3-H3K27me3_1.fastq.gz
CM-mtc91-low-iron-3-H3K27me3_CM-mtc91-low-iron-3-H3K27me3_2.fastq.gz
CM-mtc91-high-iron-1-H3K27me3_CM-mtc91-high-iron-1-H3K27me3_deeptools.bigWig
CM-mtc91-high-iron-1-H3K27me3_CM-mtc91-high-iron-1-H3K27me3_deeptools_reference-normalised.bigWig
CM-mtc91-high-iron-1-H3K27me3_CM-mtc91-high-iron-1-H3K27me3_lanceotron.bed
CM-mtc91-high-iron-2-H3K27me3_CM-mtc91-high-iron-2-H3K27me3_deeptools.bigWig
CM-mtc91-high-iron-2-H3K27me3_CM-mtc91-high-iron-2-H3K27me3_deeptools_reference-normalised.bigWig
CM-mtc91-high-iron-2-H3K27me3_CM-mtc91-high-iron-2-H3K27me3_lanceotron.bed
CM-mtc91-high-iron-3-H3K27me3_CM-mtc91-high-iron-3-H3K27me3_deeptools.bigWig
CM-mtc91-high-iron-3-H3K27me3_CM-mtc91-high-iron-3-H3K27me3_deeptools_reference-normalised.bigWig
CM-mtc91-high-iron-3-H3K27me3_CM-mtc91-high-iron-3-H3K27me3_lanceotron.bed
CM-mtc91-low-iron-1-H3K27me3_CM-mtc91-low-iron-1-H3K27me3_deeptools.bigWig
CM-mtc91-low-iron-1-H3K27me3_CM-mtc91-low-iron-1-H3K27me3_deeptools_reference-normalised.bigWig
CM-mtc91-low-iron-1-H3K27me3_CM-mtc91-low-iron-1-H3K27me3_lanceotron.bed
CM-mtc91-low-iron-2-H3K27me3_CM-mtc91-low-iron-2-H3K27me3_deeptools.bigWig
CM-mtc91-low-iron-2-H3K27me3_CM-mtc91-low-iron-2-H3K27me3_deeptools_reference-normalised.bigWig
CM-mtc91-low-iron-2-H3K27me3_CM-mtc91-low-iron-2-H3K27me3_lanceotron.bed
CM-mtc91-low-iron-3-H3K27me3_CM-mtc91-low-iron-3-H3K27me3_deeptools.bigWig
CM-mtc91-low-iron-3-H3K27me3_CM-mtc91-low-iron-3-H3K27me3_deeptools_reference-normalised.bigWig
CM-mtc91-low-iron-3-H3K27me3_CM-mtc91-low-iron-3-H3K27me3_lanceotron.bed
high-iron_deeptools_merged.bigWig
high-iron_lanceotron.bed
low-iron_deeptools_merged.bigWig
low-iron_lanceotron.bed
protocol.txt
samples_table.txt

| | |
|---|---|
| Genome browser session (e.g. UCSC) | https://genome-euro.ucsc.edu/s/meganteh/Mtc77 |

## Methodology

| | |
|---|---|
| Replicates | One replicate was completed for ChIPmentation of H3K27ac and H3K4me3<br><br>3 biological replicates were conducted for ChIPmentation of H3K27me3 |
| Sequencing depth | Mtc77_H3K27ac_1: total reads = 19973012, uniquely mapped reads = 18174120, 150bp, paired end<br>Mtc77_H3K27ac_2: total reads = 41065166, uniquely mapped reads = 37244102, 150bp, paired end<br>Mtc77_H3K4me3_1: total reads = 45383637, uniquely mapped reads = 44261116, 150bp, paired end<br>Mtc77_H3K4me3_2: total reads = 44677835, uniquely mapped reads = 43441496, 150bp, paired end<br><br>CM-mtc91-high-iron-1-H3K27me3_CM-mtc91-high-iron-1-H3K27me3: total reads = 11814811, uniquely mapped reads = 2611803, 150bp, paired end<br>CM-mtc91-high-iron-2-H3K27me3_CM-mtc91-high-iron-2-H3K27me3: total reads = 14198417, uniquely mapped reads = 5534944, 150bp, paired end<br>CM-mtc91-high-iron-3-H3K27me3_CM-mtc91-high-iron-3-H3K27me3: total reads = 26789263, uniquely mapped reads = 15278465, 150bp, paired end<br>CM-mtc91-low-iron-1-H3K27me3_CM-mtc91-low-iron-1-H3K27me3: total reads = 17195614, uniquely mapped reads = 5581204, 150bp, paired end<br>CM-mtc91-low-iron-2-H3K27me3_CM-mtc91-low-iron-2-H3K27me3: total reads = 23443092, uniquely mapped reads = 14339582, 150bp, paired end<br>CM-mtc91-low-iron-3-H3K27me3_CM-mtc91-low-iron-3-H3K27me3: total reads = 19071539, uniquely mapped reads = 7099537, 150bp, paired end |
| Antibodies | H3K27ac (Diagenode, polyclonal, C15410196), H3K4me3 (Diagenode, polyclonal, C15410003), H3K27me3 (Sigma Aldrich, polyclonal, 7-449) |
| Peak calling parameters | Read Mapping: ChIP-seq: bowtie2 with default parameters<br>Peak calling with LanceOtron: lanceotron callpeaks -c 0.5 |
| Data quality | Reads were filtered to remove PCR duplicates. LanceOtron called peaks were filtered for peaks with a peak score > 0.5. |
| Software | Data was analysed using the SeqNado analysis pipeline which can be found here: https://github.com/alsmith151/SeqNado. However, briefly: Quality control of FASTQ reads was performed using FastQC v0.12.1, adapters and poor quality bases were removed using trim_galore v0.6.10. Samples were aligned to the hg19 genome assembly using bowtie2 v2.5.1. PCR duplicates were removed using picard MarkDuplicates v3.0.0. Problematic genomic regions present in the ENCODE Blacklist (https://doi.org/10.1038/s41598-019-45839-z) were removed from the aligned files and further QC of the aligned files was performed using samtools v1.17. BigWigs were generated using the deepTools (v3.5.1) bamCoverage command 128, with the flags –extendReads –normalizeUsing RPKM. We used the deep learning based peak caller LanceOtron v1.0.8 (with a peak score cut-off value of 0.5) to call peaks. UCSC data hub generation for the BigWig and peak files was performed using a custom script. |

# Flow Cytometry

## Plots

Confirm that:

- [x] The axis labels state the marker and fluorochrome used (e.g. CD4-FITC).
- [x] The axis scales are clearly visible. Include numbers along axes only for bottom left plot of group (a 'group' is an analysis of identical markers).
- [ ] All plots are contour plots with outliers or pseudocolor plots.
- [x] A numerical value for number of cells or percentage (with statistics) is provided.

## Methodology

| | |
|---|---|
| Sample preparation | Purified cultured murine CD8+ T-cells were collected, stained using fluorescent dyes and conjugated antibodies and optionally fixed and permeabilised prior to acquisition |
| Instrument | Attune NxT flow cytometer |
| Software | Attune NxT software and FlowJo version 10.8.1-10.10.0 |
| Cell population abundance | After magnetic separation, purity of CD8+ T-cells was typically >85% as assessed by flow cytometry. By day 2 of culture, purity was typically >98%. |
| Gating strategy | Cell populations were pure (>98%) CD8+ T-cells by day 2 of culture, therefore, gating was primarily used to remove doublets and dead cells |

Scatter (FSC-A vs SSC-A, remove cells on boundaries) -> Singlets (FSC-A vs FSC-H) -> viable cells (live dead NIR negative) -> CD8+ T-cells (CD8+)

☒ Tick this box to confirm that a figure exemplifying the gating strategy is provided in the Supplementary Information.
